# Impact of home confinement due to the COVID‐19 outbreak on vitamin D levels and trends among children with pneumonia aged 1–35 months

**DOI:** 10.1002/pdi3.41

**Published:** 2023-11-05

**Authors:** Xueer Wang, Jianchuan Chen, Runting Huang, Ting Gong, Lin Zhu, Tingting Luo, Shu Yang, Li Yan, Gang Geng, Jihong Dai, Xiaoqiang Li, Daiyin Tian

**Affiliations:** ^1^ Department of Respiratory Disease Children's Hospital of Chongqing Medical University National Clinical Research Center for Child Health and Disorders Ministry of Education Key Laboratory of Child Development and Disorders Chongqing Key Laboratory of Pediatrics Chongqing China; ^2^ Chengdu Medical College Chengdu China; ^3^ School of Intelligent Medicine Chengdu University of Traditional Chinese Medicine Chengdu China; ^4^ Department of Clinical Laboratory Children's Hospital of Chongqing Medical University Chongqing China

**Keywords:** 25‐hydroxyvitamin D, children, community‐acquired pneumonia, COVID‐19, home confinement, vitamin D

## Abstract

Vitamin D plays a vital role in immunity and is related to susceptibility and the severity of pneumonia. The home confinement caused by the novel coronavirus disease (COVID‐19) decreased sunlight exposure derived from outdoor activities in children, thereby possibly exerting an influence on 25‐hydroxyvitamin D [25(OH)D] levels. The aim of this study is to quantify vitamin D level changes and trends among infants and toddlers with community‐acquired pneumonia (CAP) during and post the home confinement period. This study included children who were hospitalized in the respiratory department of the Children's Hospital of Chongqing Medical University with CAP between February 1 and July 31 from 2020 to 2022 (*N* = 397). We used propensity score matching to control the confounding bias. The levels and trends of the children's serum 25(OH)D concentrations overall and by age groups were compared between the different periods. The serum 25(OH)D concentration during the home confinement period was lower (*p* < 0.05) but was still at the vitamin D sufficiency level. There was a gradual decrease in the 25(OH)D concentrations in the subsequent several months after the beginning of home confinement, and the recovery time was delayed. When analyzed by age group, the serum 25(OH)D concentration of the toddler group changed more significantly than that of the infant group between the different periods. The insufficiency of sunlight exposure caused by home confinement resulted in a slight and gradual decrease in vitamin D levels among children with CAP. In addition, the impact was more significant for toddlers.

## Graphical abstract

The insufficiency of sunlight exposure caused by home confinement resulted in a slight and gradual decrease in vitamin D levels among children with CAP. Meanwhile, the impact was more significant for toddlers.

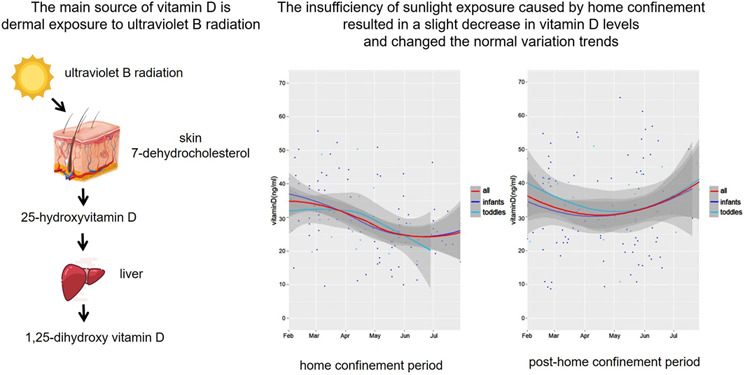

## 1 INTRODUCTION

The novel coronavirus disease‐2019 (COVID‐19) outbreak occurred in Wuhan, Hubei in December 2019, and rapidly spread worldwide. The World Health Organization declared COVID‐19 to be a pandemic on 11 March 2020.^1^ An entire set of policies aimed at diminishing viral transmission during the pandemic were implemented by the Chinese government. Chongqing initiated public health response levels I and II between January 23 and 24 March 2020 and implemented a series of non‐pharmaceutical interventions, including advice on home confinement and free movement limitation.^2^ The pandemic was controlled effectively as the virus transmission rate was slowed due to these policies. In addition, people's lifestyles changed and outdoor activities notably decreased, especially for children. A cross‐sectional repeated measures study in Hong Kong indicated markedly lower outdoor activity among children during the confinement caused by COVID‐19.^3^

Vitamin D is a type of fat‐soluble vitamin. The primary source of vitamin D is dermal exposure to ultraviolet B (UVB) radiation, contributing 80%–90% to the vitamin D replenishment in the body, while dietary sources contribute little.^4^ The UVB exposure component of sunlight (wavelengths 290–315 nm) transforms 7‐dehydrocholesterol to previtamin D3 that then isomerizes to vitamin D3 in the epidermis. After this, vitamin D3 is converted through 25‐hydroxylation into 25‐hydroxyvitamin D [25(OH)D] in the liver, which is the major form of vitamin D that circulates in the blood, providing the best assessment of the vitamin D nutritional status.^5^ Subsequently, 25(OH)D undergoes 1‐alpha‐hydroxylation to 1,25‐dihydroxy vitamin D [1,25(OH)2D] that functions through the vitamin D receptor in the nucleated cells present in various systems.^6^, ^7^

Vitamin D is best known for maintaining bone and calcium homeostasis and has been the subject of increasing interest in extra skeletal effects, especially in immunity regulation.^8^ Vitamin D possesses the ability to reduce the expression of pro‐inflammatory cytokines and increase the expression of anti‐inflammatory cytokines by macrophages.^9^ In addition, respiratory epithelial cells can convert 25(OH)D to the active form known as 1,25(OH)2D that can induce the expression of cathelicidin or CD14 by cells of the innate immune system used for antibacterial and antiviral functions.^10^ Community‐acquired pneumonia (CAP) is the major cause of childhood morbidity and hospital admission, even the leading cause of childhood mortality globally.^11^ The accumulating evidence suggests that a vitamin D deficiency may enhance the susceptibility of CAP in children and be associated with severity.^12^, ^13^

The purpose of this study is to compare the differences in vitamin D levels and trends between home confinement and post‐home confinement periods in infants and toddlers with CAP. In addition, a further aim is to analyze which age group was influenced more significantly by home confinement caused by the pandemic.

## 2 MATERIALS AND METHODS

### 2.1 Study participants and selected parameters

This study was a retrospective cohort study. The inclusion criteria were patients aged 1–35 months hospitalized in the respiratory department of the Children's Hospital of Chongqing Medical University with CAP from February 1 to July 31 from 2020 to 2022. In view of the particularity of the etiology and clinical manifestations of neonatal pneumonia, neonates were excluded from this study. CAP is defined as infectious pneumonia acquired outside the hospital (in the community), and it includes pneumonia caused by infection with a pathogen with a defined incubation period that develops after admission to a hospital. The novel coronavirus pneumonia is a special case that has been defined as a category B infectious disease in China. Hence, COVID‐19 was excluded from the criterion of CAP in this study. The definition and severity of CAP were determined according to the Guidelines for The Management of CAP in Children (2013 revised edition).^14^

According to reports published by the Chongqing Health Commission, the public health response level I and II was initiated in Chongqing and the home confinement and free movement limitations were implemented between January 23 and 24 March 2020. Subsequently, the public health response level III was initiated between March 25 and 31 August 2020, and the home confinement and free movement limitations were gradually released during this period. In addition, according to the reports published by Chongqing Health Commission, after 1 September 2020, there was no large‐scale home confinement, and movement limitation occurred in Chongqing until 31 July 2022. Thereby, we considered February 1 to July 31 in 2020 as the home confinement period, and February 1 to July 31 in 2021 and 2022 as the post‐home confinement period.

According to reports published by the Chongqing Health Commission, the public health response levels I and II were initiated in Chongqing, and home confinement and free movement limitations were implemented between January 23 and 24 March 2020. Subsequently, the public health response level III was initiated between March 25 and 31 August 2020, and home confinement and free movement limitations were gradually released during this period. In addition, according to reports published by the Chongqing Health Commission, after 12 September 2020, there was no large‐scale home confinement, and movement limitation occurred in Chongqing until 31 July 2022. Therefore, we considered February 1 to 31 July 2020 as the home confinement period, and February 1 to July 31 in 2021 and 2022 as the post‐home confinement period.

Children who had dysplasia of the airway or a history of premature birth or medical history of vitamin D‐associated metabolic disorders, such as skeletal or gastrointestinal system diseases, liver or kidney diseases, genetic syndromes, obesity, and malnutrition and malabsorption disorders, were excluded.^15^

The data of age, sex, weight, date of hospital visits, severity of CAP, and serum level of 25(OH)D stored in the hospital information system were extracted. In addition, we called the parents of the children we recruited to ask for information regarding vitamin D supplementation.

Serum 25(OH)D concentrations were determined using a chemiluminescent immunoassay. The vitamin D assay in this study was a competitive immunoassay that used anti‐fluorescein monoclonal mouse antibodies covalently bound to paramagnetic particles (PMP), anti‐25(OH)D monoclonal mouse antibodies labeled with acridinium ester, and fluorescein‐labeled vitamin D analogs. There was an inverse relationship between the 25(OH)D concentration in the patients' samples and the relative luminescence unit detected by the system, and the testing results were automatically generated by the established calibration curve. The vitamin D statuses were classified into three grades according to the guidelines developed by the American Academy of Pediatrics: Vitamin D Deficiency in Children and Its Management defines deficiency (≤15 ng/mL), insufficiency (15–20 ng/mL), and sufficiency (≥20 ng/mL).^16^

### 2.2 Data analysis

RStudio 4.1.3 was used for the statistical analysis. Descriptive statistics of the patients' characteristics were summarized using frequencies and percentages for the categorical variables, and these were compared using the chi‐squared or Fisher's exact test. The continuous variables that obeyed a normal distribution used the mean with standard deviation (SD) and were compared numerically using a Student's *t* test or variance analysis. The median with the interquartile range (IQR) was used for non‐normally distributed variables, and these were compared using a rank sum test. *p* < 0.05 were considered statistically significant. We used propensity score matching (PSM) and the nearest neighbor method to perform 1:1 matching to control the confounding bias. We divided patients aged 1–11 months old into the infant group and patients aged 12–35 months old into the toddler group according to the Child Health Care^17^ guidelines. We then compared the 25(OH)D concentrations using a Student's *t* test of all the patients, between different age groups, and between different periods. *p* < 0.05 were considered statistically significant. In addition, we plotted scatter diagrams and used a local regression to plot trendlines (confidence interval of 95%) to characterize the variation in the 25(OH)D concentrations over time of all the patients and in different age groups pre‐matched and post‐matched.

## 3 RESULT

### 3.1 Patient characteristics and basic clinical data

**TABLE 1:** The demographic details and basic clinical data before PSM.

	Home confinement period (*n* = 188)	Post‐home confinement period (*n* = 209)	*p*
Sex (*n*, %)			
Male	115 (61.2)	136 (65.1)	0.483
Female	73 (38.8)	73 (34.9)	
Age (*n*, %)			
1–11 months old	154 (81.9)	131 (62.7)	<0.001
12–35 months old	34 (18.1)	78 (37.3)	
Weight (kg)	7.57 ± 2.88	8.86 ± 2.72	<0.001
Severity of CAP (*n*, %)			
Non‐severe CAP	168 (89.4)	159 (76.1)	0.001
Severe CAP	20 (10.6)	50 (23.9)	
Gradual vitD supplement (*n*, %)			
Yes	152 (80.9)	179 (85.6)	0.252
Not	36 (19.1)	30 (14.4)	
Month of collection (*n*, %)			
Feb	58 (30.9)	27 (12.9)	<0.001
Mar	54 (28.7)	35 (16.7)	
Apr	17 (9.0)	35 (16.7)	
May	30 (16.0)	61 (29.2)	
Jun	17 (9.0)	33 (15.8)	
Jul	12 (6.4)	18 (8.6)	

*Note*: Values are presented as number (%) or mean ± standard deviation. The home confinement period was from February 1 to 31 July 2020. The post‐home confinement period was from February 1 to July 31 in 2021 and 2022.

Abbreviations: CAP, community‐acquired pneumonia; PSM, propensity score matching; vitD, vitamin D.

**TABLE 2:** The demographic details and basic clinical data after PSM.

	Home confinement period (*n* = 117)	Post‐home confinement period (*n* = 117)	*p*
Sex (*n*, %)			
Male	63 (53.8)	74 (63.2)	0.185
Female	54 (46.2)	43 (36.8)	
Age (*n*, %)			
1–11 months old	89 (76.1)	91 (77.8)	0.877
12–35 months old	28 (23.9)	26 (22.2)	
Weight (kg)	7.96 ± 3.07	8.34 ± 2.65	0.303
Severity of CAP (*n*, %)			
Non‐severe CAP	105 (89.7)	103 (88.0)	0.835
Severe CAP	12 (10.3)	14 (12.0)	
Gradual vitD supplement (*n*, %)			
Yes	96 (82.1)	99 (84.6)	0.726
Not	21 (17.9)	18 (15.4)	
Month of collection (*n*, %)			
Feb	25 (21.4)	26 (22.2)	0.911
Mar	23 (19.7)	23 (19.7)	
Apr	17 (14.5)	15 (12.8)	
May	29 (24.8)	31 (26.5)	
Jun	13 (11.1)	16 (13.7)	
Jul	10 (8.5)	6 (5.1)	

*Note*: Values are presented as number (%) or mean ± standard deviation. The home confinement period was from February 1 to 31 July 2020. The post‐home confinement period was from February 1 to July 31 in 2021 and 2022.

Abbreviations: CAP, community‐acquired pneumonia; PSM, propensity score matching; vitD, vitamin D.

Our study samples included 397 children aged 1–35 months, of which 188 were recruited during the home confinement period and 209 were recruited during the post‐home confinement period. The demographic details and basic clinical data are summarized and compared in Table 1. As can be seen in Table 1, there was a significant difference in age, weight, severity of CAP, and month of sample collection (*p* < 0.001, <0.001, 0.001, and <0.001, respectively). The disturbance of these confounding factors may have led to inaccurate results. Therefore, we used PSM and the nearest neighbor method to perform 1:1 matching, taking weight, age, severity of CAP, and month of sample collection as the matching variables to eliminate the impact of confounding bias on the results. The sample size post‐matched was 117 for the home confinement and post‐home confinement period. The demographic details and basic clinical data after PSM are summarized and compared in Table 2. As can be seen in Table 2, the above confounding factors were balanced after matching (*p* = 0.877, 0.303, 0.835, and 0.911).

### 3.2 Comparison of vitamin D levels

Table 3 shows the serum 25(OH)D concentrations for all the patients and the different age groups between the home confinement and post‐home confinement period before and after PSM, respectively. As can be seen in Table 3, the serum 25(OH)D concentration in the post‐home confinement period was higher compared to the home confinement period for all patients, including the infant and toddler groups before PSM (*p* < 0.05). However, after using PSM to eliminate the impact of confounding bias, there remained a significant difference in the overall and toddler group, but not in the infant group, of the serum 25(OH)D concentration between different periods (*p* = 0.135). Additionally, even with the use of PSM to eliminate the impact of confounding bias, the serum 25(OH)D concentrations for all patients, infants, and the toddler groups were all at the vitamin D sufficiency level.

**TABLE 3:** Serum 25(OH)D concentrations (ng/mL) between the different periods before and after PSM.

	Before PSM		*p*	After PSM		*p*
	Home confinement period	Post‐home confinement period		Home confinement period	Post‐home confinement period	
Overall	29.61 ± 10.38	33.86 ± 12.74	<0.001	29.58 ± 10.17	33.05 ± 12.66	0.022
Infants	29.44 ± 10.82	32.88 ± 13.91	0.020	29.57 ± 10.77	32.30 ± 13.45	0.135
Toddlers	30.40 ± 8.16	35.51 ± 10.38	0.012	29.59 ± 8.11	35.68 ± 9.14	0.012

*Note*: Values are presented as mean ± standard deviation. The unit of the 25(OH)D concentration is ng/mL. The infants included children aged 1–11 months and the toddlers included children aged 12–35 months. The home confinement period was from February 1 to 31 July 2020. The post‐home confinement period was from February 1 to July 31 in 2021 and 2022.

Abbreviation: PSM, propensity score matching.

Table 4 shows a comparison of the vitamin D levels between the home confinement and post‐home confinement periods before and after PSM, respectively. No significant difference was found in the proportion of vitamin D deficiency, insufficiency, and sufficiency in all age groups regardless of the use of PSM to eliminate the impact of confounding bias.

**TABLE 4:** Vitamin D levels between different periods before and after PSM.

	Before PSM		*p*	After PSM		*p*
	Home confinement period	Post‐home confinement period		Home confinement period	Post‐home confinement period	
Overall						
Deficiency	15 (7.2)	16 (8.5)	0.073	8 (6.8)	8 (6.8)	1.000
Insufficiency	14 (6.7)	15 (8.0)		10 (8.5)	10 (8.5)	
Sufficiency	180 (86.1)	157 (83.5)		99 (84.6)	99 (84.6)	
Infants						
Deficiency	13 (9.9)	16 (10.4)	0.977	8 (9.0)	7 (7.7)	0.751
Insufficiency	11 (8.4)	12 (7.8)		7 (7.9)	10 (11.0)	
Sufficiency	107 (81.7)	126 (81.8)		74 (83.1)	74 (81.3)	
Toddlers						
Deficiency	2 (2.6)	0 (0.0)	0.371^a^	0 (0.0)	1 (3.8)	0.140^a^
Insufficiency	3 (3.8)	3 (8.8)		3 (10.7)	0 (0.0)	
Sufficiency	73 (93.6)	31 (91.2)		25 (89.3)	25 (96.2)	

*Note*: Values are presented as number (%). The classification of vitamin D status is based on the serum 25(OH)D concentration: deficiency (≤15 ng/mL), insufficiency (15–20 ng/mL), and sufficiency (≥20 ng/mL). The infants included children aged 1–11 months, and the toddlers included children aged 12–35 months. The home confinement period was from February 1 to July 31 in 2020, and the post‐home confinement period was from February 1 to July 31 in 2021 and 2022.

Abbreviation: PSM, propensity score matching.

^a^ Fisher's exact test.

### 3.3 Trends in the 25(OH)D concentration over time

**FIGURE 1:**
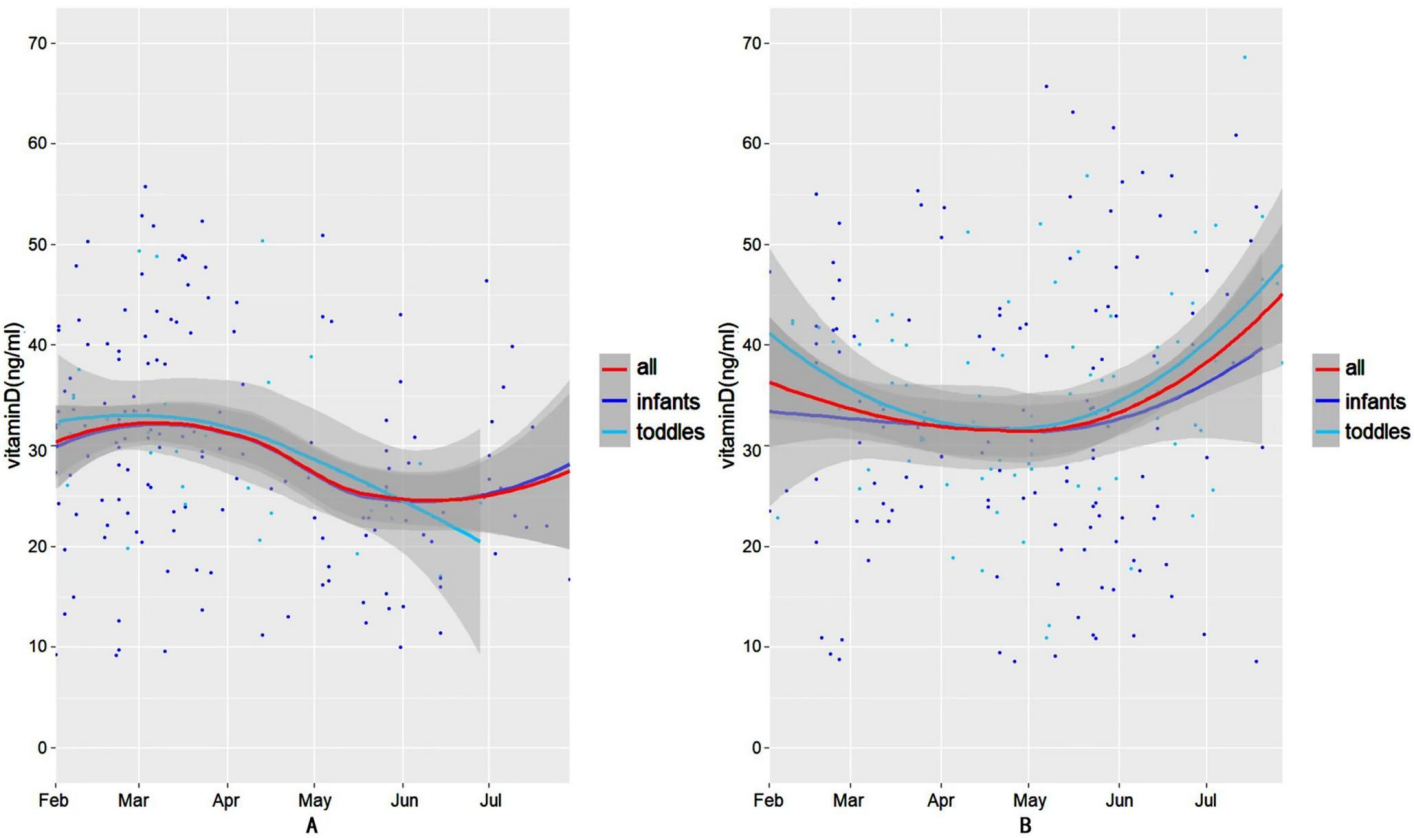
Analysis of trends over time of the serum 25(OH)D concentration before propensity score matching. (A) The trend of the serum 25(OH)D concentration during the home confinement period. (B) The trend of the serum 25(OH)D concentration during the post‐home confinement period.

**FIGURE 2:**
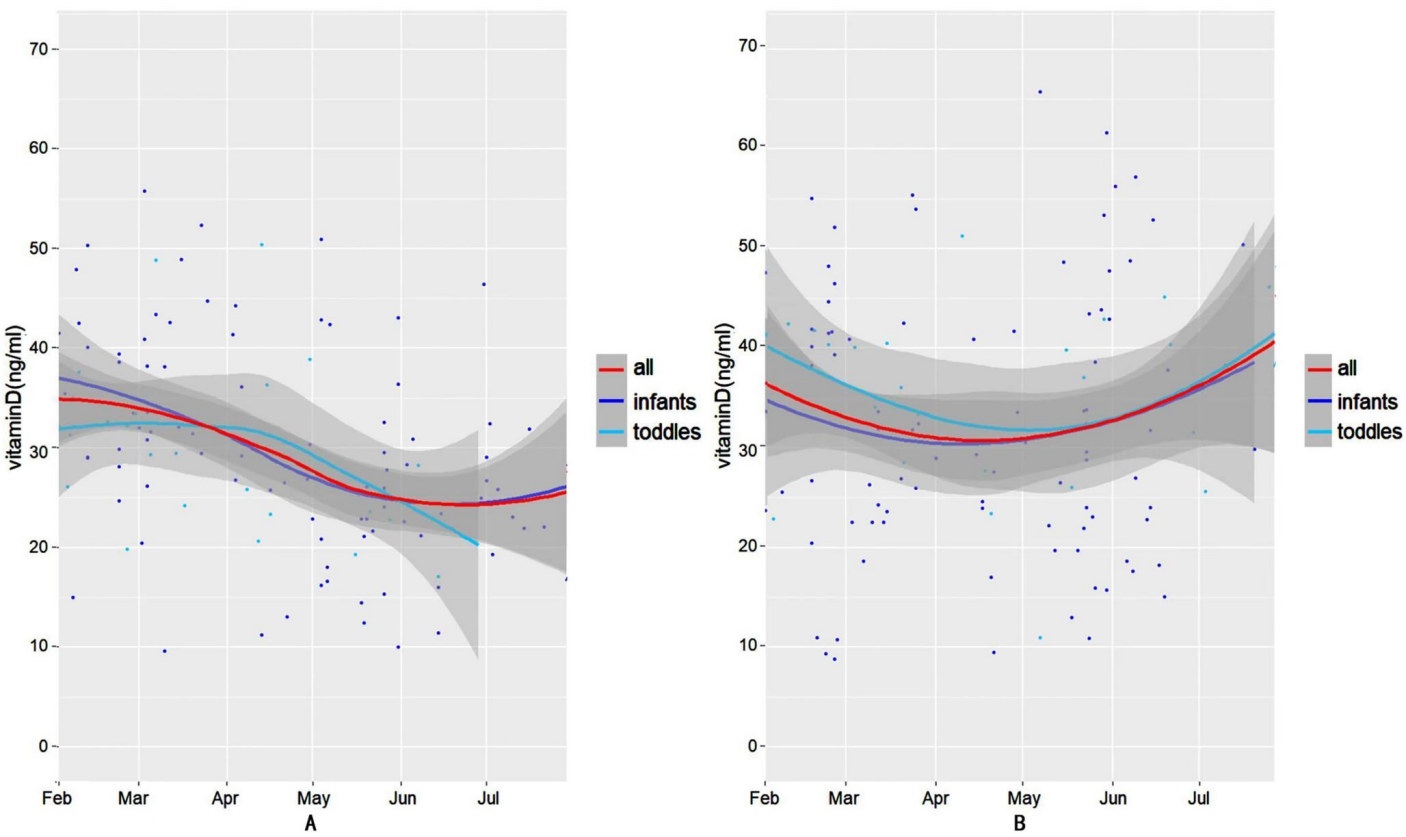
Analysis of trends over time of the serum 25(OH)D concentration after propensity score matching. (A) The trend of the serum 25(OH)D concentration during the home confinement period. (B) The trend of the serum 25(OH)D concentration during the post‐home confinement period.

Figures 1 and 2 show the 25(OH)D concentration trends over time before and after PSM in the home confinement and the post‐home confinement period, respectively. In the home confinement period, whether pre‐matched (Figure 1A) or post‐matched (Figure 2A), the serum 25(OH)D concentrations of all the patients (the red line), infants (the purple line), and the toddler (the blue line) group gradually decreased in the subsequent several months after home confinement began, but it remained at the vitamin D sufficiency level. The serum 25(OH)D concentrations of all the patients and the infant group showed a rise after July. Nevertheless, during the post‐home confinement period, there was a gradual decrease to a relatively lower level from February to April and a subsequent gradual rise after May for all patients and the different age groups before using PSM (Figure 1B).

During the post‐home confinement period, regardless of the overall, infant, and toddler group, the serum 25(OH)D concentrations gradually decreased to relatively low levels between February and April, followed by a gradual increase to higher levels beginning in May. In addition, a similar trend occurred after matching the confounding factors (Figure 2B).

## 4 DISCUSSION

The aim of this study was to evaluate the impact of the home confinement caused by the COVID‐19 pandemic on vitamin D levels and the trends among children aged 1–35 months old with CAP between the home confinement and post‐home confinement period.

As expected, the 25(OH)D concentrations decreased during the home confinement period compared to the post‐home confinement period, no matter whether there were matching confounding factors or not. Even so, the mean 25(OH)D concentrations remained at sufficient levels (≥20 ng/mL). In addition, the proportions of the vitamin D level categories did not change significantly between the two different periods. According to the vitamin D supplementation guidelines, 400–1000 IU/day should be supplied for infants up to 1 year old and 600–1000 IU/day for children over 1 year old.^18^ With the popularization of nutrition knowledge, an increasing number of parents are aware of the importance of vitamin D supplements.^19^ In our study, we collected information on patients' vitamin D supplement conditions and found that the majority of children we recruited were supplied vitamin D following the recommended dose (80.9% during the home confinement period and 85.6% during the post‐home confinement period). Moreover, telecommuting caused by the pandemic resulted in more time at home for patients, which may have increased the adherence to vitamin D supplements.^20^ In conclusion, timely and regular vitamin D supplementation may have been one of the reasons that the mean 25(OH)D concentration remained at a sufficient level, and the proportions of the vitamin D level categories did not change significantly between two different periods. Even so, a decrease in the 25(OH)D concentration was shown during the home confinement period, suggesting that outdoor activity plays a vital role in vitamin D levels, even with supplementation.

Due to the limited physical and motor capacities of infants,^21^ the opportunity for outdoor activity is less in infants than in toddlers. Furthermore, infants often have more skin covered by clothing, resulting in decreased sunlight exposure. This may have been a reason that home confinement caused by the pandemic had a greater impact on the 25(OH)D concentration in toddlers than in infants. Other studies have had similar conclusions. For example, Yu et al. found that the 25(OH)D concentration in 2020 was higher than that in 2017 and 2018 among children aged 6 months to 1 year. However, among children aged three to six, the 25(OH)D concentration in 2020 was lower than that in 2017–2019.^15^

Moreover, guidelines of various organizations and societies recommend long‐term and regular vitamin D supplements for children of all age groups.^22^, ^23^, ^24^ However, some guidelines do not emphasize that children older than 1 year must take vitamin D supplements. For example, the consensus of the Italian Pediatric Society recommends vitamin D supplementation only in children older than 1 year with risk factors for vitamin D deficiency.^25^ Also, parents tend to neglect vitamin D supplements, and supplement adherence gradually decreases as children grow. These could have been some of the reasons toddlers were affected more significantly than infants. This also suggests that vitamin D levels in toddlers require more attention, especially when outdoor activities decrease.

Because the majority of serum 25(OH)D is produced by dermal tissue through sunlight exposure, the serum 25(OH)D concentration gradually increases with enhanced ultraviolet B radiation due to seasonal changes.^26^, ^27^ Satoshi et al. found that the serum 25(OH)D concentration was significantly higher in summer and autumn than in winter and spring among healthy children up to 48 months old.^28^ Similarly, in our study, there was a gradual decrease before May, and a rise occurred in the subsequent months when the UVB radiation increased because of seasonal variation during the post‐home confinement period. However, during the home confinement period, the seasonal variation in the serum 25(OH)D concentration changed. Among the patients, infants, and the toddler group, the serum 25(OH)D concentrations gradually decreased in the subsequent months after home confinement began, and it did not begin to rise in May and even continued to fall until July. In agreement with our study, a study conducted in Hong Kong described the trend of serum 25(OH)D concentrations in children aged 2–24 months from June 2019 to November 2020 using an interrupted time series analysis and showed a similar result.^29^ Due to the lipophilic nature of vitamin D3 [the precursor of 25(OH)D], it can be stored in adipose tissue for several months, and the half‐life is approximately 2 months,^30^ which may be relevant in the gradual decline of the serum 25(OH)D concentration whether due to the seasonal factor or reduced outdoor activities. Additionally, it has been suggested the hysteresis of decreases and deficiencies in vitamin D levels indicate that it may be necessary to monitor vitamin D levels regularly for children with risk factors for vitamin D deficiency. In addition, this may develop into guidance for the follow‐up of vitamin D supplementation therapy.

This study has the following limitations. First, our study obtained data from a single‐center. Due to the small sample size and uneven year distribution, instead of comparing the samples of 2021 and 2022 separately to 2020, we combined them into one group. Second, our data lacked information of weather related to differences in sunlight exposure during different periods and the participants' dietary information, including the dietary structure and duration of breastfeeding. In addition, a current study suggests that eating habits changed because of COVID‐19 confinement among children.^31^ These factors might have resulted in biased estimates. Finally, we did not collect the serum 25(OH)D concentration of healthy children in the same period, so we cannot be sure whether the decrease in vitamin D levels caused by home confinement led to the increased susceptibility to pneumonia in children. Further research is required.

## 5 CONCLUSION

In this study, it was found that Vitamin D played a vital role in growth, bone mineralization, and immunomodulation during early childhood and was related to the susceptibility and severity of pneumonia. Even though vitamin D was supplied for the majority of infants and toddlers, the home confinement caused by the COVID‐19 outbreak led to a slight and gradual decrease in vitamin D levels among children with CAP, especially for toddlers. These findings could inform future policy for preventive vitamin D supplementation among infants and toddlers under similar conditions, such as limitations of outdoor activities and sunlight exposure.

## AUTHOR CONTRIBUTIONS

**Xueer Wang**: Conceptualization, data curation, investigation, formal analysis, writing‐original draft, writing – review & editing. **Jianchuan Chen**: Conceptualization, data curation, resources, project administration. **Runting Huang**: Formal analysis, methodology, visualization. **Ting Gong**: Formal analysis, visualization. **Lin Zhu**: Formal analysis, visualization. **Tingting Luo**: Data curation. **Shu Yang**: Methodology. **Li Yan**: resources. **Gang Geng**: Resources. **Jihong Dai**: Writing – review & editing. **Xiaoqiang Li**: Resources, supervision. **Daiyin Tian**: Conceptualization, project administration, supervision, validation, writing – review & editing.

## CONFLICT OF INTEREST STATEMENT

The authors declare that the research was conducted without any commercial or financial relationships that could be construed as a potential conflict of interest.

## ETHICS STATEMENT

The study protocol was approved by the Ethics Committee of Children's Hospital of Chongqing Medical University (File No. (2022)435).

## Data Availability

The data that support the findings of this study are available from the corresponding author upon reasonable request.
